# Epileptic seizure detection with deep EEG features by convolutional neural network and shallow classifiers

**DOI:** 10.3389/fnins.2023.1145526

**Published:** 2023-05-22

**Authors:** Wei Zeng, Liangmin Shan, Bo Su, Shaoyi Du

**Affiliations:** ^1^School of Physics and Mechanical and Electrical Engineering, Longyan University, Longyan, China; ^2^School of Mechanical Engineering and Automation, Fuzhou University, Fuzhou, China; ^3^Institute of Artificial Intelligence and Robotics, Xi'an Jiaotong University, Xi'an, China

**Keywords:** electroencephalogram (EEG), epileptic seizure detection, deep features, shallow classifiers, deep neural network (DNN), convolution neural network

## Abstract

**Introduction:**

In the clinical setting, it becomes increasingly important to detect epileptic seizures automatically since it could significantly reduce the burden for the care of patients suffering from intractable epilepsy. Electroencephalography (EEG) signals record the brain's electrical activity and contain rich information about brain dysfunction. As a non-invasive and inexpensive tool for detecting epileptic seizures, visual evaluation of EEG recordings is labor-intensive and subjective and requires significant improvement.

**Methods:**

This study aims to develop a new approach to recognize seizures automatically using EEG recordings. During feature extraction of EEG input from raw data, we construct a new deep neural network (DNN) model. Deep feature maps derived from layers placed hierarchically in a convolution neural network are put into different kinds of shallow classifiers to detect the anomaly. Feature maps are reduced in dimensionality using Principal Component Analysis (PCA).

**Results:**

By analyzing the EEG Epilepsy dataset and the Bonn dataset for epilepsy, we conclude that our proposed method is both effective and robust. These datasets vary significantly in the acquisition of data, the formulation of clinical protocols, and the storage of digital information, making processing and analysis challenging. On both datasets, extensive experiments are performed using a cross-validation by 10 folds strategy to demonstrate approximately 100% accuracy for binary and multi-category classification.

**Discussion:**

In addition to demonstrating that our methodology outperforms other up-to-date approaches, the results of this study also suggest that it can be applied in clinical practice as well.

## 1. Introduction

Epileptic seizures are brain's electrical activities that occurs suddenly and unexpectedly (Arab et al., 2010). It affects the daily life of more than 50 million individuals in the world due to the brain dysfunction (Solaija et al., 2018). The recurrent epileptic seizure usually occurs without any obvious external symptoms (Zhou et al., 2020). Currently, using metal electrodes fixed to the brain scalp in a standard configuration, electroencephalogram (EEG) signals record neural activity. Physiologically, they offer deep insight into the brain's state and can be used to detect seizure onsets non-invasively and economically. Traditionally, clinical diagnosis relies on the visual screening and inspection of pronged EEG recordings by board-certified physicians, which is cumbersome, subjective and error-prone (Martis et al., 2015). A reliable, efficient, and accurate EEG analysis and classification system is therefore urgently needed to detect seizures in a timely manner. To handle this problem, different tools have been developed and applied rapidly in recent years, including signal processing and artificial intelligence (Gupta et al., 2018; Li et al., 2019; Subasi et al., 2019; Shoeibi et al., 2021; Tuncer et al., 2021a).

Detection of seizures using EEG generally involves two phases: separating features and classifying them. In the first phase, numerous features generated from four domains, including time, frequency, time-frequency, and non-linear, are incorporated. To analyse time-domain characteristics, morphological parameters, including duration, amplitude, kurtosis, and peak are representative (Wang et al., 2020). There is widespread use of fast Fourier transform (Li and Chen, 2021), as well as power spectral density in frequency domain analysis, provided the EEG signal is static (Al Ghayab et al., 2018). The EEG signal, however, does not display stationarity. Hence, methods of time-frequency domain analysis are usually used for the analysis o time-varying properties of the EEG signal (Sharma et al., 2020), such as time-frequency distribution (Wu et al., 2021) and wavelet transform (Tuncer et al., 2021b). In wavelet transforms, relative frequency information, which is present at low frequencies as well as relative time information, is captured at high frequencies via multiresolution analysis (Sharmila and Geethanjali, 2019). In addition to the wavelet transform, other variations have been proposed, such as the empirical wavelet transform, wavelet packet transform, and wavelet packet entropy. Another popular approach to extracting features is the empirical mode decomposition (EMD) in combination with its variants (Li et al., 2021). Intrinsic mode functions (IMFs) are created when the EEG signal is broken into subsignals. Nonetheless, EMD cannot handle multi-channel signals. Cura and Akan (2021) proposed a single- and multi-channel EEG-based dynamic pattern decomposition (DMD) method to analyze epileptic signals. They extracted high-order spectral moments and subband powers to detect seizure. In non-linear domain, complexity metrics are proposed to depict chaotic properties of the EEG signal, like Hurst exponent, Lyapunov exponent, and various entropies. Other kinds of non-linear metrics, such as Lempel-Ziv complexity, have also been widely used. Rout et al. (2021) used variational mode decomposition (VMD) to identify three band-limited eigenmode functions (BLIMFs) in EEG raw data. In order to derive information-rich spectral and temporal features from BLIMFs, the Hilbert Transform was applied. In addition, the most discriminatory compressed form of privileged information was analyzed based on approximate entropy (ApEn). Anuragi et al. (2022) employed EWT to break down the EEG recordings into Fourier Bessel Series Expansion (FBSE) based subbands. These subbands were then reconstructed as a three-dimensional (3D) phase space representation (PSR). An Euclidean distance of the 3D PSR was used in order to calculate features like line length, log energy entropy, and norm energy entropy. Shankar et al. (2021) used a recurrence plot (RP) technique to analyze brain rhythms with two-dimensional images generated from the EEG signal, which could preserve the non-linear characteristics of EEG. As an additional assessment of image quality, RP entropy and root mean square skewness were used along with RP image criteria.

In the second phase, a variety of machine learning algorithms were proposed to extract EEG signal features, such as artificial neural networks and logistic regression (Abbasi and Goldenholz, 2019; Beniczky et al., 2021). EEG signals during seizures were differentiated using DWT and arithmetic coding by Amin et al. (2020). Various classifiers were then used to detect seizure activity, including Naïve Bayes (NB), multi-layer perceptron (MLP), k nearest neighbors (KNN), and support vector machine (SVM). Anter et al. (2022) utilize a NB based hybrid genetic whale optimization algorithm for feature selection. Afterwards, the ictal and non-ictal EEG signals were classified using an adaptive ELM based on a differential evolution algorithm. To separate EEG signals into distinct bands, Shoeibi et al. (2022) used TQWT. Then, 13 different types of fuzzy entropies were calculated as features from different subbands. Afterwards, EEG recordings were separated using an adaptive neuro-fuzzy inference system.

Due to rapid development in deep learning (DL) over the past few years, several emerging algorithms have been utilized to handle seizure detection problems. While building a multi-layer neural network, DL approaches can minimize the impact of irrelevant features and alleviate computation costs. Acharya et al. (2018) developed a multi-layer deep convolutional neural network (CNN) to determine whether a patient was in a normal, preictal, or seizure state. At present, the generalization and classification abilities of existing DL models may be limited by the use of inter-layer static connection weights. To overcome such problems, A new network architecture called Variable Weight Convolutional Neural Networks (VWCNN) was proposed by Jia et al. (2022). In its convolutional and fully-connected layers, dynamic weights were used instead of static weights to adapt to different EEG characteristics. This model could handle a variety of situations. Sahani et al. (2021) used modified particle swarm optimization based on log energy entropy maxima to calculate optimized values. Then, epileptic seizures were detected using a combination of multiple complex deep neural networks.

Among machine learning systems, representative features have often been hand-designed and empirically chosen. Such systems are more likely to produce false positives and are prone to misdiagnosis. By contrast, DL automatically generates features instead of using any hard-crafted features, and have the potential to provide superior classification performance (Murat et al., 2021). These techniques automate feature extraction and no manual feature extraction is required due to the end-to-end structure of DL models. In this work, we build an efficient and reliable deep neural network (DNN) to recognize epilepsy, utilizing features from CNN layers without any preprocessing of input EEG signals. This study makes a major contribution to the identification of presence and developing stages of seizure using information from deep feature maps of CNN together with shallow classifiers. An effective way for reduction of the dimensionality of deep feature maps is the employment of Principal Component Analysis (PCA) (Jolliffe and Cadima, 2016).

Throughout the article, the following structures are followed. The proposed method is described in detail in Section 2, which includes description of EEG data, extraction of deep feature, and EEG classification for seizure detection. Section 3 designs comprehensive experiments and provides corresponding results. Section 4 presents a comprehensive discussion about the results and contribution. Section 5 gives a brief conclusion.

## 2. Materials and methods

**Figure 1:**
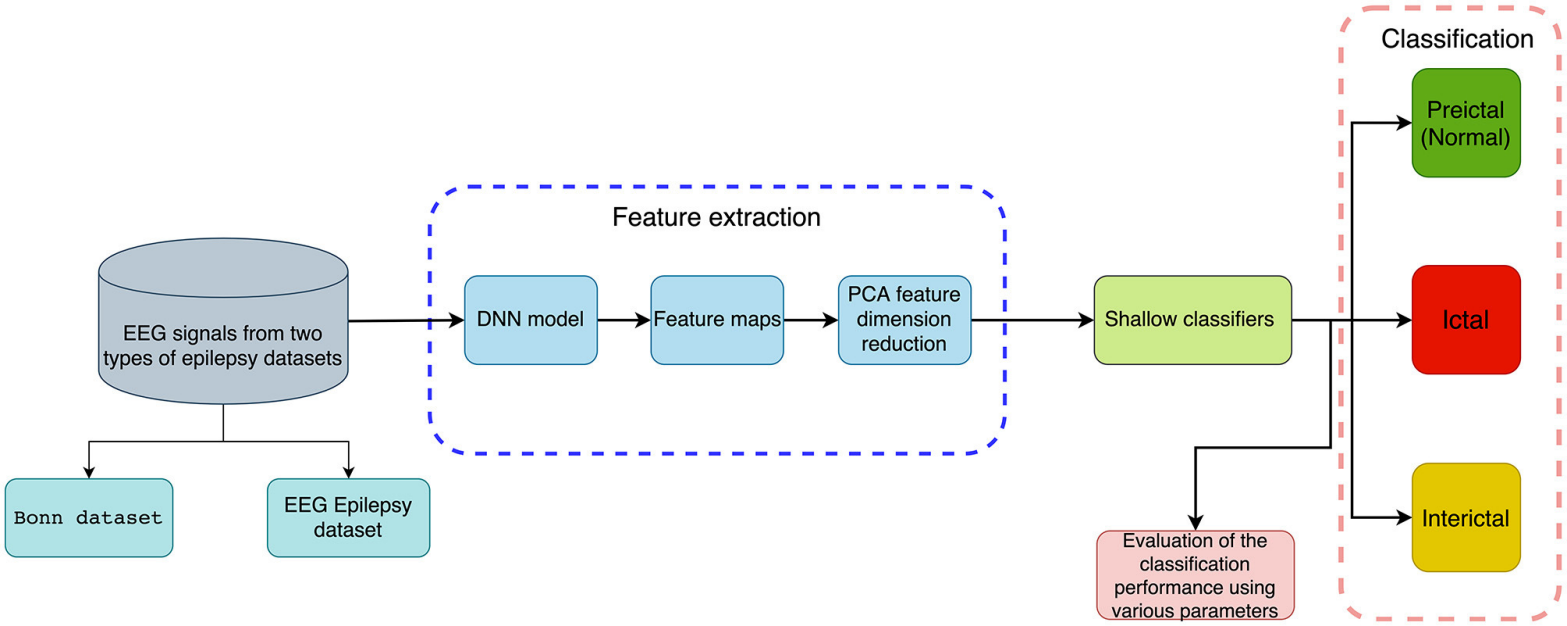
An illustration of the proposed method for classifying EEG recordings with deep features and shallow classifiers for the detection of epileptic seizures (binary and multi-class classification).

This section briefly introduces a method for distinguishing normal and abnormal EEG signals with information extracted through deep features for detecting epileptic seizures. It consists of a feature extraction phase and a classification phase, which includes several steps. Firstly, EEG recordings are subjected to DNN-based feature extraction without any preprocessing, followed by PCA reduction of feature dimension. Secondly, features are put into five traditional machine learning classifiers to detect epileptic seizures. It includes binary classification (seizure vs. seizure-free or preictal vs. interictal) and multi-class classification (preictal vs. interictal vs. ictal). A flowchart showing our method is available in Figure 1.

### 2.1. EEG database

#### 2.1.1. Dataset-1

**Figure 2:**
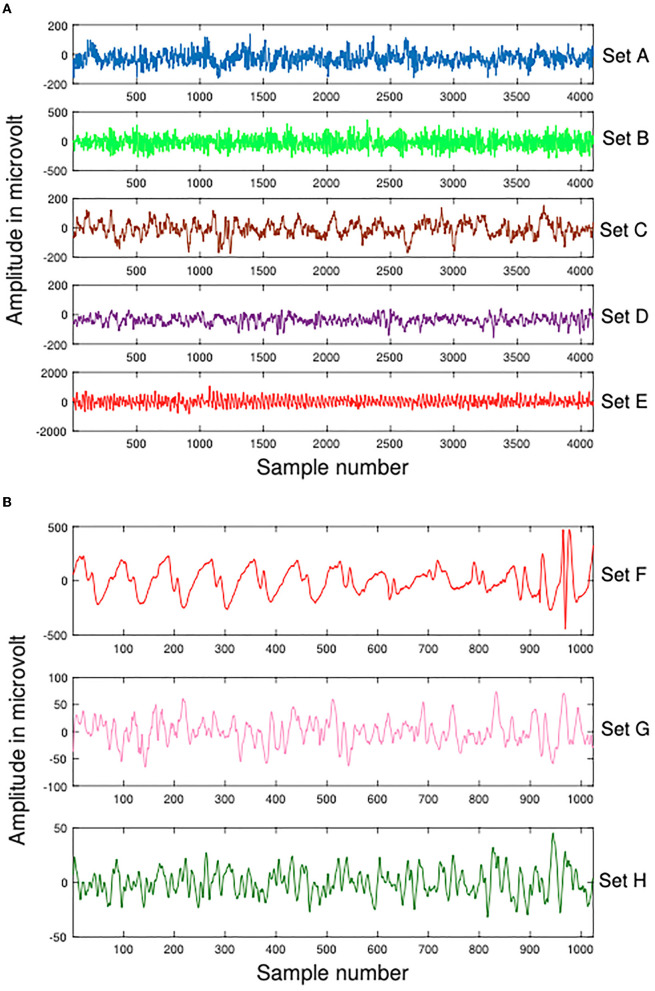
Samples of Dataset-1 Bonn dataset and Dataset-2 EEG Epilepsy dataset. **(A)** Dataset-1 Bonn dataset A, B, C, D, and E. **(B)** Dataset-2 EEG Epilepsy dataset F, G, and H.

A part of the experimental data for this study comes from the Bonn dataset, which is publicly available (Andrzejak et al., 2001). Each subset of the dataset contains 100 artifact-free, single-channel intracranial EEG clips of 23.6 s each, labeled A, B, C, D, and E (also Z, O, N, F, and S, accordingly). An amplifier system with 128 channels and a band-pass filter between 0.53 and 40 Hz was used to record the EEG signals at 173.61 Hz. Therefore, each signal contains 4,097 records, that is, each signal has a data length of 4,097. These data are demonstrated in Figure 2. Table 1 summarizes details about this dataset.

**Table 1:** Overview of Dataset-1.

**Items**	**Set A**	**Set B**	**Set C**	**Set D**	**Set E**
Participants	5 healthy controls	5 healthy controls	5 epileptic patients	5 epileptic patients	5 epileptic patients
Electrode types	Scalp	Scalp	Intracranial	Intracranial	Intracranial
Participants' states	Awake with opened eyes	Awake with closed eyes	Interictal	Interical	Ictal
Total number of epochs	100	100	100	100	100
Sampling rate (Hz)	173.61	173.61	173.61	173.61	173.61
Duration of each epoch (second)	23.6	23.6	23.6	23.6	23.6

#### 2.1.2. Dataset-2

In Dateset-2, segmented EEG recordings were obtained from 10 epilepsy patients (Swami et al., 2016). With a GrassTelefactor Comet AS40 amplifier system and a 200 Hz sampling rate, all EEG recordings were acquired. The duration of each EEG recording is approximately 5.12 s (1,024 samples). These data are demonstrated in Figure 2. The scalp electrodes for EEG recordings were gold-plated and adhered to the 10-20 standard in compliance with the recording procedure. First, an EEG signal was filtered with a bandpass filter having a cutoff frequency of 0.5 and 70 Hz. Afterwards, It was divided by clinical experts into ictal (group F), interictal (group G), and preictal (group H) phases. Table 2 summarizes details about this dataset.

**Table 2:** Overview of Dataset-2.

**Items**	**Set F**	**Set G**	**Set H**
Participants	10 epilepsy patients	10 epilepsy patients	10 epilepsy patients
Electrode types	Scalp	Scalp	Scalp
Participants' states	Ictal	Interictal	Preictal (normal)
Total number of epochs	50	50	50
Sampling rate (Hz)	200	200	200
Duration of each epoch (second)	5.12	5.12	5.12

### 2.2. Deep feature extraction

**Figure 3:**
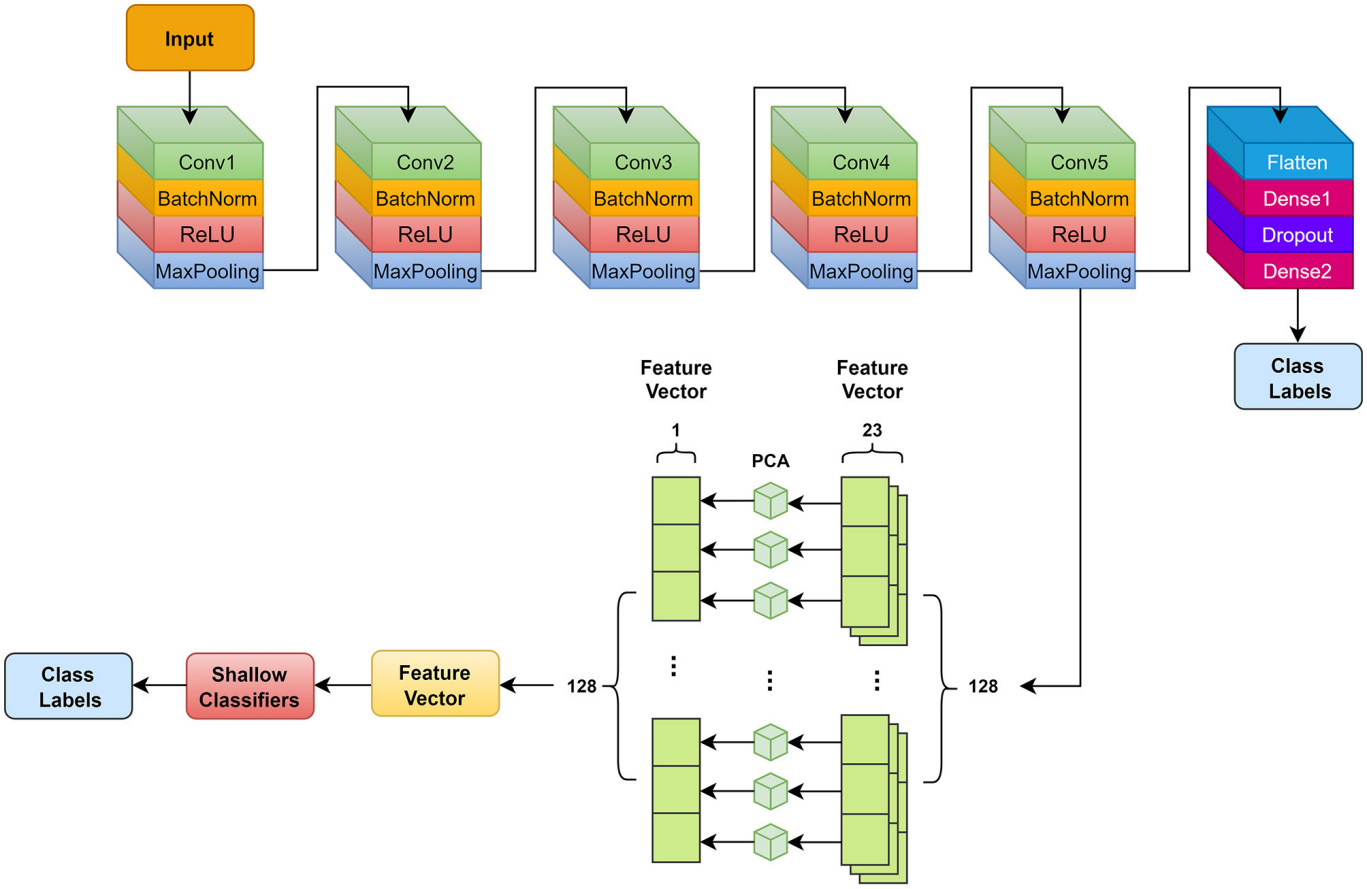
Deep neural network model and feature extraction used in this study. Conv, convolution.

DL techniques learn a set of empirical features at multiple abstraction levels, capable of learning complex functions through input data independent of hand-crafted features. It undergoes a learning process by progressively extracting multiple features from low layers to high layers (Murat et al., 2021). Therefore, we use the DNN-based model to automatically generate features. Figure 3 demonstrates this DNN-based model.

Our DNN model outputs feature maps after we have connected the convolutional layer. PCA is used to remove useless features and reduce redundancy, which can alleviate the computational cost and enhance the performance and generalization. Figure 3 demonstrates the feature extraction steps and details.

Table 3 summarizes a detailed parameter representation of the DNN model. We add a Batch Normalization (BatchNorm) layer after each convolutional layer, with axis 2 and momentum 0.9 to speed up training. An activation function for rectified linear unit (ReLU) follows each BatchNorm layer. We use *L*_2_ regularization to alleviate overfitting with a dropout of 0.4 upon reaching the first fully connected layer. Aggregate data are used for subject-level assessments. Our neural network weights are updated by using the cross-entropy loss function and Adam optimization. There are three settings: 0.0001, 50, and 300, which are the learning rate, batch size, and epochs. A 0.001 learning rate is applied to the data, a batch size of 50, and 300 epochs are used when training the model. A feature map sized 23 × 128 is exported from the MaxPooling layer ahead of the flatten layer. We split the eigenvectors into 128 small eigenvectors of shape size 23 × 1. PCA is then used to perform dimensionality reduction on each of the small eigenvectors, resulting in 128 eigenvectors of shape size 1 × 1. These feature vectors are concatenated with 1 × 128 shape size and fed into shallow classifiers below for classification.

**Table 3:** Model summary of DNN.

**No**	**Layer name**	**Layer parameters**	**Output shape**	**Number of params**
1	1D Convolution	Filters = 32, kernel_size = 3, input_shape = (4097,1), stride = 1, padding = “valid”	(4095,32)	128
2	BatchNorm	Axis = 2, momentum = 0.9	(4095,32)	128
3	Activation	ReLU	(4095,32)	0
4	1D MaxPooling	Pool_size = 2 stride = 2 padding = “valid”	(2047,32)	0
5	1D Convolution	Filters = 64, kernel_size = 5, stride = 1, padding = “valid”	(2043,64)	10,304
6	BatchNorm	Axis = 2, momentum = 0.9	(2043,64)	256
7	Activation	ReLU	(2043,64)	0
8	1D MaxPooling	Pool_size = 4, stride = 4, padding = “valid”	(510,64)	0
9	1D Convolution	Filters = 128, kernel_size=13, stride = 1, padding = “valid”	(498,128)	106,624
10	BatchNorm	Axis = 2, momentum = 0.9	(498,128)	512
11	Activation	ReLU	(498,128)	0
12	1D MaxPooling	Pool_size = 4, stride = 4, padding = “valid”	(124,128)	0
13	1D Convolution	Filters = 256, kernel_size = 17, stride = 1, padding = “valid”	(108,256)	557,312
14	BatchNorm	Axis = 2, momentum = 0.9	(108,256)	1,024
15	Activation	ReLU	(108,256)	0
16	1D MaxPooling	Pool_size = 2, stride = 2, padding = “valid”	(54,256)	0
17	1D Convolution	Filters = 128, kernel_size = 9, stride = 1, padding = “valid”	(46,128)	295,040
18	BatchNorm	Axis = 2, momentum = 0.9	(46,128)	512
19	Activation	ReLU	(46,128)	0
20	1D MaxPooling	Pool_size = 2, stride = 2, padding = “valid”	(23,128)	0
21	Flatten	-	2,944	0
22	Dense	Unit = 64, activation = “ReLU”, kernel_regularizer = *L*_2_ (0.03)	64	188,480
23	Dropout	Rate = 0.4	64	0
24	Dense	Unit = 5, activation = “softmax”	5	325

### 2.3. Machine learning classifiers

For epileptic seizure detection, in addition to support vector classifier (SVC) (Lau and Wu, 2003), several classical machine learning classifiers are employed, including k-nearest neighbors (KNN) (Kramer, 2013), gradient boosting (GB) (Natekin and Knoll, 2013), random forest (RF) (Lau and Scornet, 2016), Gaussian Naïve Bayes (GNB) (Griffis et al., 2016), decision tree (DT) (Safavian and Landgrebe, 1991), and multi-layer perception (MLP) (Murtagh, 1991). Shallow classifiers are still the classifier of choice despite deep learning approaches becoming increasingly overwhelming. To solve supervised classification problems, discriminant analysis is utilized to reduce the distance between each class and increase the variability between different classes (Ye et al., 2004; Murat et al., 2021).

## 3. Results

We design comprehensive experiments on two databases and illustrate the results of classifying EEG categories into binary and multi-class. The DNN model is implemented in TensorFlow backend using a 10-core Intel Core i9 CPU and RTX3090 GPU on a high-performance computer.

Cross-validation using a *K*-fold (*K* = 10) method verifies the effectiveness of the classification. Each iteration will use *K*−1 folds to train and the remaining folds to test. In addition to accuracy (ACC), specificity (SPF), and sensitivity (SEN), we use another four classic performance indicators: negative predictive value (NPV), positive predictive value (PPV), F1 score, and Matthews correlation coefficient (MCC). Here is the calculation: a True Positive is equal to TP, a False Negative is equal to FN, a True Negative is equal to TN, and a False Positive is equal to FP. For larger MCC value, the classifier performs better.


(1) $$\begin{array}{l} \text{SEN}=\frac{\text{TP}}{\text{TP}+\text{FN}}\times 100\left(\text{\%}\right) \end{array}$$

(2) $$\begin{array}{l} \text{SPF}=\frac{\text{TN}}{\text{TN}+\text{FP}}\times 100\left(\text{\%}\right) \end{array}$$

(3) $$\begin{array}{l} \text{ACC}=\frac{\text{TP}+\text{TN}}{\text{TP}+\text{TN}+\text{FN}+\text{FP}}\times 100\left(\text{\%}\right) \end{array}$$

(4) $$\begin{array}{l} \text{PPV}=\frac{\text{TP}}{\text{TP}+\text{FP}}\times 100\left(\text{\%}\right) \end{array}$$

(5) $$\begin{array}{l} \text{NPV}=\frac{\text{TN}}{\text{TN}+\text{FN}}\times 100\left(\text{\%}\right) \end{array}$$

(6) $$\begin{array}{l} \text{MCC}=\frac{\text{TP}\times \text{TN}-\text{FN}\times \text{FP}}{\sqrt{\left(\text{TP}+\text{FN}\right)\left(\text{TP}+\text{FP}\right)\left(\text{TN}+\text{FN}\right)\left(\text{TN}+\text{FP}\right)}}\times 100\left(\text{\%}\right) \end{array}$$

(7) $$\begin{array}{l} \text{F}1\text{ score}=\frac{2\times \text{TP}}{2\times \text{TP}+\text{FN}+\text{FP}}\times 100\left(\text{\%}\right) \end{array}$$

Table 4 shows the comprehensive experiments setting. Using the Bonn and EEG Epilepsy datasets, twelve and five different classification problems are proposed, respectively. They focus on differentiating between preictal (normal), interictal, and ictal EEG signals, including binary and multi-class classification.

**Table 4:** Different experimental cases for Bonn and EEG Epilepsy datasets.

**Dataset**	**Case**	**Groups**	**Description**				
			**Type 1**	**Type 2**	**Type 3**	**Type 4**	**Type 5**
Bonn	1	A vs. E	Normal (eyes open)	Ictal	-	-	-
	2	B vs. E	Normal (eyes closed)	Ictal	-	-	-
	3	AB vs. E	Normal	Ictal	-	-	-
	4	C vs. E	Interictal	Ictal	-	-	-
	5	D vs. E	Interictal	Ictal	-	-	-
	6	CD vs. E	Interictal	Ictal	-	-	-
	7	A vs. D	Normal	Interictal	-	-	-
	8	ABCD vs. E	Non-seizure	Seizure	-	-	-
	9	AB vs. CDE	Normal	Epileptic	-	-	-
	10	A vs. C vs. E	Normal	Interitcal	Ictal	-	-
	11	AB vs. CD vs. E	Normal	Interitcal	Ictal	-	-
	12	A vs. B vs. C vs. D vs. E	Normal (eyes open)	Normal (eyes closed)	Interictal	Interictal	Ictal
EEG Epilepsy	I	F vs. G	Ictal	Interictal	-		
	II	F vs. H	Ictal	Preictal	-		
	III	G vs. H	Interictal	Preictal	-		
	IV	F vs. GH	Seizure	Seizure-free	-		
	V	F vs. G vs. H	Ictal	Interictal	Preictal		

**Figure 4:**
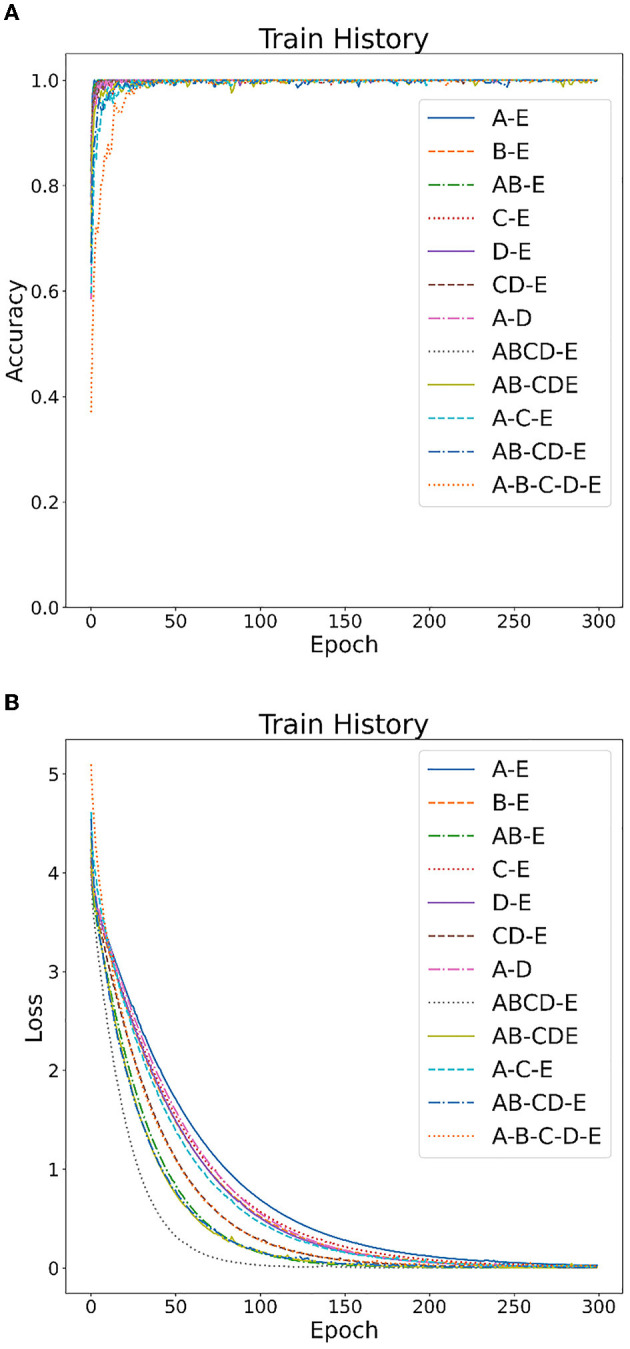
CNN model training on Bonn dataset: **(A)** accuracy curve, **(B)** loss curve.

**Figure 5:**
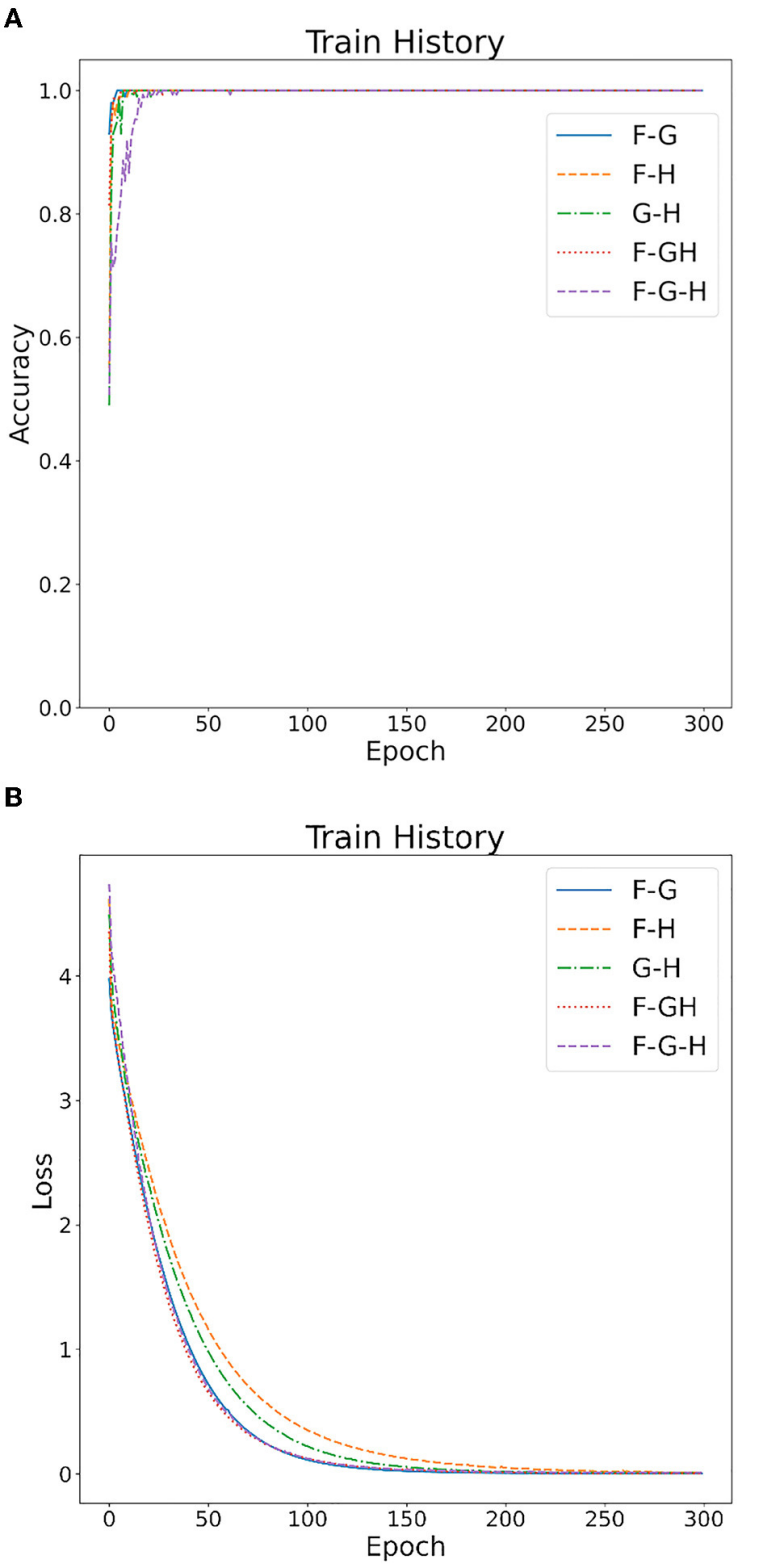
CNN model training on EEG Epilepsy dataset: **(A)** accuracy curve, **(B)** loss curve.

Figures 4, 5 show the overall accuracy and loss curves for the model trained on two datasets. It is obvious that after almost 300 epochs the network converges.

**Figure 6:**
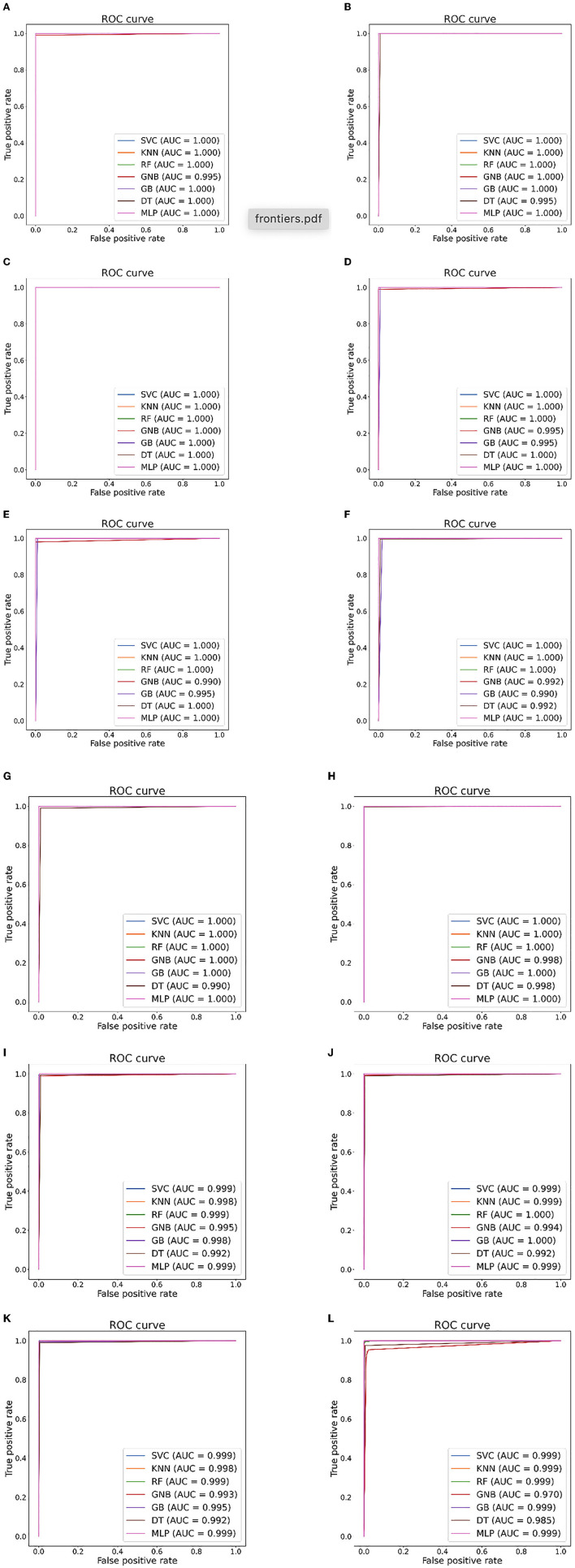
Seizure detection ROC curves and AUC from Bonn dataset. **(A)** Case 1: A-E. **(B)** Case 2: B-E. **(C)** Case 3: AB-E. **(D)** Case 4: C-E. **(E)** Case 5: D-E. **(F)** Case 6: CD-E. **(G)** Case 7: A-D. **(H)** Case 8: ABCD-E. **(I)** Case 9: AB-CDE. **(J)** Case 10: A-C-E. **(K)** Case 11: AB-CD-E. **(L)** Case 12: A-B-C-D-E.

**Figure 7:**
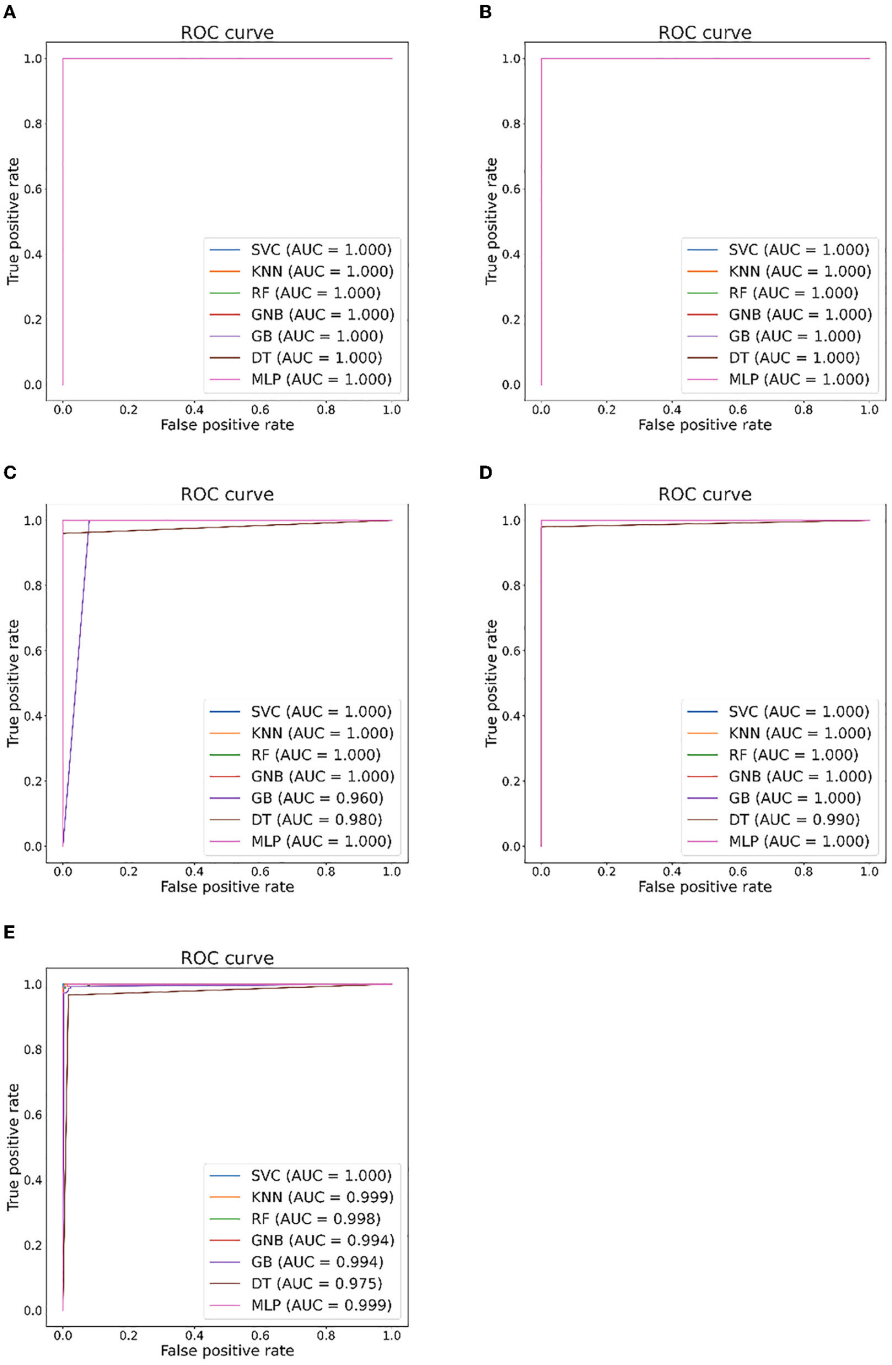
Seizure detection ROC curves and AUC from EEG Epilepsy dataset. **(A)** Case I: F-G. **(B)** Case II: F-H. **(C)** Case III: G-H. **(D)** Case IV: F-G. **(E)** Case V: F-G-H.

Tables 5, 6 illustrate the classification results for different cases on two datasets, respectively. To further illustrate the performance of each shallow classifier, Figures 6, 7 show the ROC curves and associated AUC for 12 cases of the Bonn dataset and 5 cases of the EEG epilepsy dataset. Our study demonstrates improved accuracy in discriminating between preictal, interictal, and ictal EEG signals. As a whole, the proposed method performs well and yields good results, demonstrating that it can distinguish various classes of EEG signals effectively.

**Table 5:** The evaluation of the performance of the proposed approach using 10-fold cross-validation style on Bonn dataset with 12 cases.

**Case**	**Classifier**	**ACC (*%*)**	**SPF (*%*)**	**SEN (*%*)**	**PPV (*%*)**	**NPV (*%*)**	**MCC (*%*)**	**F1 (*%*)**
Case 1	SVC	100	100	100	100	100	100	100
	KNN	100	100	100	100	100	100	100
	RF	100	100	100	100	100	100	100
	GNB	99.50	100	99.00	100	99.00	99.00	99.49
	GB	100	100	100	100	100	100	100
	DT	100	100	100	100	100	100	100
	MLP	100	100	100	100	100	100	100
Case 2	SVC	100	100	100	100	100	100	100
	KNN	100	100	100	100	100	100	100
	RF	100	100	100	100	100	100	100
	GNB	100	100	100	100	100	100	100
	GB	100	100	100	100	100	100	100
	DT	99.50	99.00	100	99.00	100	99.00	99.50
	MLP	100	100	100	100	100	100	100
Case 3	SVC	100	100	100	100	100	100	100
	KNN	100	100	100	100	100	100	100
	RF	100	100	100	100	100	100	100
	GNB	100	100	100	100	100	100	100
	GB	100	100	100	100	100	100	100
	DT	100	100	100	100	100	100	100
	MLP	100	100	100	100	100	100	100
Case 4	SVC	100	100	100	100	100	100	100
	KNN	100	100	100	100	100	100	100
	RF	100	100	100	100	100	100	100
	GNB	99.50	100	99.00	100	99.00	99.00	99.49
	GB	99.50	99.00	100	99.00	100	99.00	99.50
	DT	100	100	100	100	100	100	100
	MLP	100	100	100	100	100	100	100
Case 5	SVC	99.50	100	99.00	100	99.00	99.00	99.49
	KNN	100	100	100	100	100	100	100
	RF	100	100	100	100	100	100	100
	GNB	99.00	100	98.00	100	98.03	98.01	98.98
	GB	99.50	99.00	100	99.00	100	99.00	99.50
	DT	100	100	100	100	100	100	100
	MLP	100	100	100	100	100	100	100
Case 6	SVC	99.66	100	99.50	100	99.00	99.25	99.74
	KNN	100	100	100	100	100	100	100
	RF	99.66	99.00	100	99.50	100	99.25	99.75
	GNB	99.33	99.00	99.50	99.50	99.00	98.49	99.50
	GB	99.33	98.00	100	99.00	100	98.50	99.50
	DT	99.33	99.00	99.50	99.50	99.00	98.49	99.50
	MLP	100	100	100	100	100	100	100
Case 7	SVC	100	100	100	100	100	100	100
	KNN	100	100	100	100	100	100	100
	RF	100	100	100	100	100	100	100
	GNB	100	100	100	100	100	100	100
	GB	99.50	99.00	100	99.00	100	99.00	99.50
	DT	99.00	99.00	99.00	99.00	99.00	98.00	99.00
	MLP	100	100	100	100	100	100	100
Case 8	SVC	99.80	100	99.75	100	99.00	99.37	99.87
	KNN	100	100	100	100	100	100	100
	RF	100	100	100	100	100	100	100
	GNB	99.80	100	99.75	100	99.00	99.37	99.87
	GB	99.80	99.00	100	99.75	100	99.37	99.87
	DT	99.80	100	99.75	100	99.00	99.37	99.87
	MLP	99.80	100	99.75	100	99.00	99.37	99.87
Case 9	SVC	99.60	99.66	99.50	99.50	99.66	99.16	99.50
	KNN	99.60	99.66	99.50	99.50	99.66	99.16	99.50
	RF	99.80	99.66	100	99.50	100	99.58	99.75
	GNB	99.60	100	99.00	100	99.33	99.16	99.49
	GB	99.80	99.66	100	99.50	100	99.58	99.75
	DT	99.20	99.00	99.50	98.51	99.66	98.33	99.00
	MLP	99.60	99.66	99.50	99.50	99.66	99.16	99.50
Case 10	SVC	99.66	99.83	99.66	99.66	99.83	99.49	99.66
	KNN	99.66	99.83	99.66	99.66	99.83	99.49	99.66
	RF	100	100	100	100	100	100	100
	GNB	99.33	99.66	99.33	99.33	99.66	98.99	99.33
	GB	100	100	100	100	100	100	100
	DT	99.00	99.50	99.00	99.00	99.50	98.49	99.00
	MLP	99.66	99.83	99.66	99.66	99.83	99.49	99.66
Case 11	SVC	99.80	99.90	99.80	99.80	99.90	99.70	99.80
	KNN	99.80	99.90	99.80	99.80	99.90	99.70	99.80
	RF	99.60	99.80	99.60	99.60	99.80	99.40	99.60
	GNB	99.20	99.60	99.20	99.20	99.60	98.80	99.20
	GB	99.20	99.60	99.20	99.20	99.60	98.80	99.20
	DT	99.00	99.50	99.00	99.00	99.50	98.50	99.00
	MLP	99.80	99.90	99.80	99.80	99.90	99.70	99.80
Case 12	SVC	99.80	99.95	99.80	99.80	99.95	99.74	99.80
	KNN	98.80	99.70	98.80	98.80	99.70	98.49	98.80
	RF	98.80	99.70	98.80	98.80	99.70	98.49	98.80
	GNB	93.60	98.40	93.60	93.60	98.40	91.99	93.60
	GB	99.60	99.90	99.60	99.60	99.90	99.49	99.60
	DT	97.60	99.40	97.60	97.60	99.40	96.99	97.60
	MLP	99.80	99.95	99.80	99.80	99.95	99.74	99.80

**Table 6:** An evaluation of the performance of the proposed approach using 10-fold cross-validation with 5 cases of EEG epilepsy dataset.

**Case**	**Classifier**	**ACC (*%*)**	**SPF (*%*)**	**SEN (*%*)**	**PPV (*%*)**	**NPV (*%*)**	**MCC (*%*)**	**F1 (*%*)**
Case I	SVC	100	100	100	100	100	100	100
	KNN	100	100	100	100	100	100	100
	RF	100	100	100	100	100	100	100
	GNB	100	100	100	100	100	100	100
	GB	100	100	100	100	100	100	100
	DT	100	100	100	100	100	100	100
	MLP	100	100	100	100	100	100	100
Case II	SVC	100	100	100	100	100	100	100
	KNN	100	100	100	100	100	100	100
	RF	100	100	100	100	100	100	100
	GNB	100	100	100	100	100	100	100
	GB	100	100	100	100	100	100	100
	DT	100	100	100	100	100	100	100
	MLP	100	100	100	100	100	100	100
Case III	SVC	100	100	100	100	100	100	100
	KNN	100	100	100	100	100	100	100
	RF	100	100	100	100	100	100	100
	GNB	100	100	100	100	100	100	100
	GB	96.00	92.00	100	92.59	100	92.29	96.15
	DT	98.00	100	96.00	100	96.15	96.07	97.95
	MLP	100	100	100	100	100	100	100
Case IV	SVC	100	100	100	100	100	100	100
	KNN	100	100	100	100	100	100	100
	RF	100	100	100	100	100	100	100
	GNB	100	100	100	100	100	100	100
	GB	100	100	100	100	100	100	100
	DT	99.33	100	98.00	100	99.00	98.50	98.98
	MLP	100	100	100	100	100	100	100
Case V	SVC	100	100	100	100	100	100	100
	KNN	99.33	99.66	99.33	99.33	99.66	98.99	99.33
	RF	98.66	99.33	98.66	98.66	99.33	97.99	98.66
	GNB	99.33	99.66	99.33	99.33	99.66	98.99	99.33
	GB	97.33	98.66	97.33	97.33	98.66	95.99	97.33
	DT	96.66	98.33	96.66	96.66	98.33	94.99	96.66
	MLP	99.33	99.66	99.33	99.33	99.66	98.99	99.33

## 4. Discussion

The seizure detection literature shows that several methods are currently available to handle binary and multi-category classification issues. Experimental results for 17 epilepsy detection cases have been presented and discussed in detail. A comparison of our algorithm with other up-to-date solutions is provided in Table 7.

**Table 7:** Summary of literature comparison results (10-fold cross-validation style).

**References**	**Methodology**	**Cases**	**ACC (*%*)**	**Our ACC (*%*)**
Cetin et al. (2015)	Autoregressive coefficients with BP + Elman neural networks	A-E	98.3	100
Jiang et al. (2020)	Symplectic geometry eigenvalues + SVM	A-E	100	100
Subasi et al. (2019)	GA, PSO and SVM	A-E	99.38	100
Supriya et al. (2021)	MG, EWF and AWD + SVM	A-E	100	100
Prabhakar and Lee (2022)	K-SVD, SOM + ELM, deep learning, transfer learning	A-E	98.35	100
Swami et al. (2016)	DT-CWT + GRNN	B-E	98.9	100
Ahmedt-Aristizabal et al. (2018)	End-to-end data and RNNs + LSTM	B-E	94.75	100
Jiang et al. (2020)	Symplectic geometry eigenvalues + SVM	B-E	99.33	100
Supriya et al. (2021)	MG, EWF and AWD + SVM	B-E	100	100
Prabhakar and Lee (2022)	K-SVD, SOM + ELM, deep learning, transfer learning	B-E	97.57	100
Swami et al. (2016)	DT-CWT + GRNN	AB-E	99.2	100
Sharma et al. (2017)	ATFFWT + fractal dimension + LS-SVM	AB-E	100	100
Jiang et al. (2020)	Symplectic geometry eigenvalues + SVM	AB-E	100	100
Prabhakar and Lee (2022)	K-SVD, SOM + ELM, deep learning, transfer learning	AB-E	97.84	100
Swami et al. (2016)	DT-CWT + GRNN	C-E	98.7	100
Sharma et al. (2017)	ATFFWT + fractal dimension + LS-SVM	C-E	99	100
Raghu et al. (2019)	Matrix determinant feature + MLP classifier	C-E	97.60	100
Jiang et al. (2020)	Symplectic geometry eigenvalues + SVM	C-E	99.33	100
Supriya et al. (2021)	MG, EWF and AWD + SVM	C-E	100	100
Swami et al. (2016)	DT-CWT + GRNN	D-E	93.3	100
Sharma et al. (2017)	ATFFWT + fractal dimension + LS-SVM	D-E	98.5	100
Raghu et al. (2019)	Matrix determinant feature + MLP classifier	D-E	97.60	100
Jiang et al. (2020)	Symplectic geometry eigenvalues + SVM	D-E	100	100
Supriya et al. (2021)	MG, EWF and AWD + SVM	D-E	100	100
Swami et al. (2016)	DT-CWT + GRNN	CD-E	95.2	100
Sharma et al. (2017)	ATFFWT + fractal dimension + LS-SVM	CD-E	98.67	100
Raghu et al. (2019)	Matrix determinant feature + MLP classifier	CD-E	96.85	100
Jiang et al. (2020)	Symplectic geometry eigenvalues + SVM	CD-E	99.28	100
Gupta et al. (2018)	DCT, Hurst exponent and ARMA + SVM	A-D	98.4	100
Tuncer et al. (2019)	Local senary pattern + SVM	A-D	99.5	100
Hassan et al. (2020)	CEEMDAN + Adaptive Boosting	ABCD-E	99.2	100
Mursalin et al. (2017)	ICFS + RF classifier	ABCD-E	97.4	100
Jiang et al. (2020)	Symplectic geometry eigenvalues + SVM	ABCD-E	99.97	100
Peng et al. (2021)	Stein kernel-based SR	AB-CDE	98.2	99.80
Acharya et al. (2018)	13-layer CNN without performing feature extraction and selection	AB-CDE	88.7	99.80
Jiang et al. (2020)	Symplectic geometry eigenvalues + SVM	AB-CDE	99.17	99.80
Jaiswal and Banka (2017)	LNDP and 1D-LGP + ANN	A-C-E	98.22	100
Gupta and Banka (2019)	WMRPE, rhythms of FBE + LS-SVM	A-C-E	97.3	100
Jiang et al. (2020)	Symplectic geometry eigenvalues + SVM	A-C-E	99.22	100
Zhang et al. (2021)	FSWT-based subbands and CSoS, FuzzyEn, HFD, t-SNE + KNN	A-C-E	99.69	100
Bhardwaj et al. (2016)	EMD + Constructive Genetic Programming	AB-CD-E	98.33	99.80
Peker et al. (2015)	DT-CWT + CVANN	AB-CD-E	97.79	99.80
Raghu et al. (2019)	Matrix determinant feature + MLP classifier	AB-CD-E	96.5	99.80
Jiang et al. (2020)	Symplectic geometry eigenvalues + SVM	AB-CD-E	99.80	99.80
Zarei and Asl (2021)	DWT and OMP + SVM	AB-CD-E	99.33	99.80
Sharma et al. (2020)	ToC + deep neural network	A-B-C-D-E	97.2	99.80
Zahra et al. (2017)	MVEMD + ANN	A-B-C-D-E	87.2	99.80
Zhang et al. (2021)	FSWT-based subbands and CSoS, FuzzyEn, HFD, t-SNE + KNN	A-B-C-D-E	93.62	99.80
Zhou et al. (2020)	SSA + SVM, ELM and ANN	F-G	94	100
Peng et al. (2021)	Stein kernel-based SR	F-G	98.00	100
Wang et al. (2021)	TVAR-MWBF-UROFR + SVM	F-G	98.18	100
Sukriti et al. (2021)	EMD-MSPCA, RCMSE, RCMFE, RCMPE + SVM	F-G	96.38	100
Tajmirriahi and Amini (2021)	SDE + SVM	F-G	99.1	100
Zhou et al. (2020)	SSA + SVM, ELM and ANN	F-H	95	100
Peng et al. (2021)	Stein kernel-based SR	F-H	99	100
Wang et al. (2021)	TVAR-MWBF-UROFR + SVM	F-H	100	100
Sukriti et al. (2021)	EMD-MSPCA, RCMSE, RCMFE, RCMPE + SVM	F-H	100	100
Tajmirriahi and Amini (2021)	SDE + SVM	F-H	96.8	100
Zhou et al. (2020)	SSA + SVM, ELM and ANN	G-H	93	100
Wang et al. (2021)	TVAR-MWBF-UROFR + SVM	G-H	88.95	100
Sukriti et al. (2021)	EMD-MSPCA, RCMSE, RCMFE, RCMPE + SVM	G-H	97.15	100
Tajmirriahi and Amini (2021)	SDE + SVM	G-H	91.5	100
Zhou et al. (2020)	SSA + SVM, ELM and ANN	F-GH	91	100
Peng et al. (2021)	Stein kernel-based SR	F-GH	97.5	100
Wang et al. (2021)	TVAR-MWBF-UROFR + SVM	F-GH	98.08	100
Peng et al. (2021)	Stein kernel-based SR	F-G-H	97.21	100
Sukriti et al. (2021)	EMD-MSPCA, RCMSE, RCMFE, RCMPE + SVM	F-G-H	93.49	100

For Bonn dataset, the first three cases handle binary classification. Regarding Case 1 (A-E), when using the EEG spectrum as input, Cetin et al. (2015) calculated autoregressive coefficients, which were then fed into back propagation (BP) and Elman neural networks. A 98.3% accuracy rate was reported as the best. Jiang et al. (2020) used a symplectic geometric decomposition method to derive features from EEG signals and put them into an SVM for EEG classification. It was reported that the accuracy was 100%. In an attempt to find the optimal parameters of an SVM to classify epileptic EEG, a mixture model was constructed using genetic algorithms (GA), as well as particle swarm optimization (PSO) by Subasi et al. (2019). A 99.38% accuracy rate was reported as the best. Using New Weighted Complex Networks (NWCNs), Supriya et al. (2021) extracted three features from EEG data: Modular Gain (MG), Average Weighted Degree (AWD), and Edge Weight Fluctuation (EWF). Three features' separation performance was examined using an SVM model with three different kernels. They obtained 100% classification accuracy. Prabhakar and Lee (2022) employed K-singular value decomposition (K-SVD) to derive sparse descriptions from EEG signals and extracted features using self-organizing maps (SOMs). The data was then fed into ELM, deep learning, and transfer learning models for classification, with an accuracy rate of 98.35%. Unlike previous methods, ours is 100% accurate.

According to Swami et al. (2016), a dual-tree complex wavelet transform (DT-CWT) was employed to divide EEG recordings into multiple subbands on a six-level scale in Case 2 (B-E). These subbands acted as features and classified EEG signals with a general regressive neural network (GRNN). A 98.9% accuracy rate was reported as the best. Ahmedt-Aristizabal et al. (2018) achieved 94.75% accuracy by using a recurrent neural network (RNN) embedded an LSTM network. Jiang et al. (2020), Supriya et al. (2021), and Prabhakar and Lee (2022) also studied on this classification issue and reported 99.33, 100, and 97.57% accuracies, respectively. Our method, on the other hand, achieves 100% accuracy.

Regarding Case 3 (AB-E), EEG clips are divided into two types: non-ictal and ictal. It was reported that Swami et al. (2016) had an accuracy rate of 99.2%. Sharma et al. (2017) used analytic time-frequency flexible wavelet transform (ATFFWT) and fractal dimensions to export features and put them into a least squares support vector machine (LS-SVM). Afterwards, a 100% accuracy rate was reported as the best. Jiang et al. (2020) and Prabhakar and Lee (2022) also studied on this classification issue and reported 100 and 97.84% accuracies, respectively. Our method, on the other hand, achieves 100% accuracy.

Regarding Cases 4 through 6, EEG signals are divided into interictal and ictal types (C-E, D-E, and CD-E). It was reported that Swami et al. (2016) had 98.7, 93.3, and 95.2% accuracies. Sharma et al. (2017) indicated 99, 98.5, and 98.67% accuracy rates. In Raghu et al. (2019), descriptive and bivariate histogram analysis, and polar histogram were used to provide matrix determinant features. The effectiveness was verified on three cases, using the MLP classifier to achieve accuracies of 97.60, 97.60, and 96.85%, respectively. Jiang et al. (2020) also studied on these issues and reported accuracies of 99.33, 100, and 99.28%, respectively. In contrast, our proposed method achieves 100, 100, and 100% accuracies, respectively.

Case 7 (A-D) addresses the classification of normal vs. interictal. Gupta et al. (2018) utilized discrete cosine transform (DCT) to build a multirate filterbank structure, which decomposed EEG signals into their respective brain rhythms. Then, the Hurst exponent together with the autoregressive moving average (ARMA) parameters were derived from the statistical results of the brain rhythms as features. The SVM classifier reported an accuracy of 98.4%. Using Local Military Patterns (LSPs), Tuncer et al. (2019) extracted binary features through EEG signals. A standard deviation based strategy was used to deal with threshold value problems of ternary functions. Then, extracted features were put into SVM for classification with an accuracy rate of 99.5%. Unlike previous methods, ours is 100% accurate.

In Case 8, the EEG is classified as seizure or non-seizure (ABCD-E). An objective method of identifying intrinsic modes was proposed in Hassan et al. (2020) by using complete ensemble empirical mode decomposition with adaptive noise (CEEMDAN). Modeling these mode functions with normal inverse Gaussian (NIG) parameters follows. They employed Adaptive Boosting to classify EEG signals and reported 99.2% accuracy. For feature derivation, Mursalin et al. (2017) examined an improved correlation-based feature selection method (ICFS). A 97.4% accuracy rate was reported for an RF classifier. Jiang et al. (2020) also focused on this issue and a 99.97% accuracy rate was reported as the best. Our method, on the other hand, achieves 100% accuracy.

Regarding Case 9 (AB-CDE), EEG signals are divided into normal and epileptic types. In Peng et al. (2021), EEG signals were classified in symmetric positive definite (SPD) matrix spaces by using Stein kernel-based sparse representations (SR). They reported accuracy rate of 98.20%. Acharya et al. (2018) developed a multiple-layer CNN algorithm to avoid feature extraction and selection. They reported 88.7% accuracy. Jiang et al. (2020) studied on this classification issue with an accuracy of 99.17%. Unlike previous methods, ours is 99.8% accurate.

In Cases 10 and 11, ternary classification is addressed by both A-C-E and AB-CD-E. We report 100% and 99.80% classification accuracies, respectively. To deal with Case 10, in Jaiswal and Banka (2017), the Local Neighborhood Description Pattern (LNDP) together with the 1D Local Gradient Pattern (1D-LGP) was utilized to export features. An adaptive neural network (ANN) was designed for classification, reporting 98.22% accuracy. Gupta and Banka (2019) achieved feature extraction of rhythms based on a combination of Weighted Multiscale Renyi Permutation Entropy (WMRPE) and Fourier-Bessel Series Expansion (FBSE). To classify these features, LS-SVM was used, and the best accuracy rate was 97.3%. Zhang et al. (2021) proposed a fusion method for feature extraction based on Frequency Sliced Wavelet Transform (FSWT). Then, these feature were fed into a KNN classifier with a classification accuracy of 99.69%. Regarding Case 11, according to Bhardwaj et al. (2016), EEG recordings were split into multiple IMFs, each with a set of bandwidth parameters extracted. They constructed genetic programming for classification and a 98.33% accuracy rate was reported as the best. Peker et al. (2015) used DT-CWT to extract features from EEG signals. EEG data was classified using a complex-valued adaptive neural network (CVANN) and a 97.79% accuracy rate was reported. In Raghu et al. (2019), a 96.5% accuracy rate was reported as the best. Jiang et al. (2020) studied on these classification issues with reported accuracies of 99.22 and 99.80%, respectively. Zarei and Asl (2021) exported different coefficients from EEG signals using DWT and Orthogonal Matching Pursuit (OMP) techniques. Then, some non-linear features and several statistical features were computed using DWT and OMP coefficients. They were put into an SVM classifier, which reported 99.33% accuracy.

In Case 12, the EEG is separated into five categories (A-B-C-D-E). Sharma et al. (2020) used third-order cumulants (ToC) to export features from EEG recording and put them into deep neural networks for classification, reporting 97.2% accuracy. In Zahra et al. (2017), using the MVEMD algorithm, the EEG recordings were decomposed into multiple intrinsic scales. An ANN model was created to classify valid IMFs with a reported accuracy of 87.2%. Zhang et al. (2021) reported 93.62% accuracy. In contrast, our proposed method achieves 99.80% accuracy.

For EEG Epilepsy dataset, Cases I to IV deal with binary classification. Zhou et al. (2020) decomposed the EEG recordings into singular values using singular spectrum analysis (SSA). Then, the log-normalized function values are calculated, forming the eigenvector. They were fed into shallow classifiers, including SVM, ELM, and ANN, to perform with the highest accuracy of 94, 95, 93, and 91% in the four cases. Wang et al. (2021) proposed an autoregressive (AR) model based time-varying (TV) modeling framework to describe EEG recordings. The multiwavelet basis function expansion (MWBF) method was used to approximate the TV parameters of the AR model (TVAR). Afterwards, the resulting extended model was reduced and refined using the Ultra-regularized Orthogonal Regression (UROFR) algorithm. The SVM achieved the highest accuracies of 98.18, 100, 88.95, and 98.08% for the four cases, respectively. Peng et al. (2021) also dealt with Cases I, II and IV and reported accuracies of 98.00, 99, and 97.5%, respectively. The EMD-MSPCA method, developed by Sukriti et al. (2021), combined empirical mode decomposition with multiscale PCA, to denoise EEG recordings. Following that, three complexity measures were used as features. DT, LDA, SVM, and KNN shallow classifiers were used for classification of Cases I, II, and III. The documented accuracy for each is 96.38, 100, and 97.15%. Due to its inherent self-similarity, Tajmirriahi and Amini (2021) used stochastic differential equations (SDEs) to model EEG signals with self-similar fractional Levy stabilization processes. They Fit the probability distribution to the derived EEG signal histogram, and extracted the parameters of the fitted histogram. A SVM classifier was used to classify them, with 99.1, 96.8, and 91.5% accuracies for cases I, II, and III, respectively. In contrast, our approach reports 100, 100, 100, and 100% accuracies for the four cases, respectively.

Case V address ternary classification. Peng et al. (2021) and Sukriti et al. (2021) reported accuracies of 97.21 and 93.49%, respectively. We report the accuracy of 100%, which also outperforms other approaches.

Unlike the aforementioned algorithms, this study designs an DNN model to automatically extract deep features from layer outputs during raining. Afterwards, extracted features are filtered by PCA for dimensionality reduction and directly put into seven shallow classifiers to classify EEG signals. The process is simple, high efficient along with high accuracy. Table 7 illustrates the comparison results on the classification performance between our approaches and other approaches recently proposed. Our method illustrates superior performance and has potential for serving as an adjunct to fMRI in epilepsy diagnosis.

Our experimental results have indicated that the proposed method is highly accurate in detecting epilepsy for binary, three-class, and five-class classification problems, illustrating the suitability of our scheme for solving problems involving multiple classes. The clinical potential of automated analysis of epileptic seizure activity is significant. Additionally, once high-performance computers are utilized, its computational simplicity is enhanced, allowing it to be deployed in clinical applications. As a result, this new approach is better equipped to satisfy clinical demands in terms of efficiency, functionality, universality, and simplicity, while providing satisfactory accuracy. These traits make it an appealing alternative option for clinical diagnosis. Real-time seizure detection for smart healthcare and Internet of Medical Things (IoMT) applications is a potential use case for the proposed method.

## 5. Conclusion

This study uses different kinds of machine learning classifiers to detect seizure with features derived from the max pooling layers of a DNN model. The suggested algorithm separates EEG recordings into two, three and five classes. The results show that performance of the advised classifier is promising for seizure detection. This model may provide neurologists with additional assistance when diagnosing epilepsy. The work in the future will incorporate a number of handcrafted features (such as intrinsic fuzzy entropy, Lyapunov exponent, and Lempel-Ziv complexity) as well as deep features to design deep learning models and compare them with current model performance. In conclusion, the proposed protocol will speed up epilepsy diagnosis, assist clinicians to implement clinical epilepsy monitoring devices with less burden.

## Data availability statement

The original contributions presented in the study are included in the article/supplementary material, further inquiries can be directed to the corresponding author.

## Ethics statement

Ethical review and approval was not required for the study on human participants in accordance with the local legislation and institutional requirements. The patients/participants provided their written informed consent to participate in this study.

## Author contributions

WZ and SD contributed to the study concept and design. WZ, LS, and BS performed the experiments and data analysis and prepared the draft manuscript. All authors participated manuscript organization and approved the submitted version.
